# The Effect of Annealing and Optical Radiation Treatment on Graphene Resonators

**DOI:** 10.3390/nano12152725

**Published:** 2022-08-08

**Authors:** Yujian Liu, Cheng Li, Shangchun Fan, Xuefeng Song, Zhen Wan

**Affiliations:** 1 School of Instrumentation Science and Opto-Electronics Engineering, Beihang University, Beijing 100191, China; 2 Research Institute of Beihang University in Shenzhen, Shenzhen 518000, China; 3 Shenzhen Institute for Quantum Science and Engineering, Southern University of Science and Technology, Shenzhen 518055, China

**Keywords:** graphene resonator, interface stress, film thermal damage, thermal time constant

## Abstract

Graphene resonant sensors have shown strong competitiveness with respect to sensitivity and size. To advance the applications of graphene resonant sensors, the damage behaviors of graphene harmonic oscillators after thermal annealing and laser irradiation were investigated by morphology analysis and frequency domain vibration characteristics. The interface stress was proven to be the key factor that directly affected the yield of resonators. The resulting phenomenon could be improved by appropriately controlling the annealing temperature and size of resonators, thereby achieving membrane intactness of up to 96.4%. However, micro-cracks were found on the graphene sheets when continuous wave (CW) laser power was more than 4 mW. Moreover, the fluctuating light energy would also cause mechanical fatigue in addition to the photothermal effect, and the threshold damage power for the sinusoidally modulated laser was merely 2 mW. In this way, based on the amplitude-frequency surface morphology of the graphene resonator, the thermal time constant of the order of a few microseconds was confirmed to evaluate the damage of the graphene oscillator in situ and in real time, which could be further extended for those resonators using other 2D materials.

## 1. Introduction

Micro resonant sensors have been widely applied in aviation, aerospace engineering and automation due to their high sensitivity, stable performance and direct frequency signal output. The resonator is the key element of resonant sensors, which dominantly affects the performance of the whole system. To develop a high-performance resonant sensor, the material of the micro resonators should be stiff, robust and stable.

Graphene, an atom-thick two-dimensional material with a single-layer thickness of 0.335 nm, has demonstrated excellent mechanical [1], optical [2], electrical [3] and thermal properties [4]. These superior properties enable the new material with novel nanostructures to be widely applied in the field of micro-electro-mechanical systems (MEMS) or photoelectric devices [5]. To be specific, graphene exhibits a high Young’s modulus of 1.0 TPa, a high tensile rate up to 20% [6] and extreme fatigue life of more than 10^9^ cycles [7], which makes it an appropriate material for harmonic oscillators. Particularly, the first graphene resonator was developed by transferring graphene onto the trenches of silicon oxide [8], actuated by a modulated laser. Compared with the silicon counterpart, a graphene resonant sensor could reach 45 times higher pressure sensitivity with a 25 times smaller membrane area [9]. However, in terms of stability, the graphene resonators still cannot reach the same long-term stability as silicon resonators that can achieve a one-year frequency drift of merely 0.01% [10]. This perfect stability of silicon resonators is not only due to the craftsmanship of silicon resonators—for example, the complete sealing technology of the resonator—but also the research of the damage resource, and the compensation methods [11,12,13]. Thus, research about the damage mechanism of graphene resonators is of vital importance, but it has not been extensively discussed thus far. Moreover, it should be noted that, especially for a merely ~0.335 nm thick membrane, it is much easier to lose integrity.

The damage of graphene resonators can be attributed to the manufacturing process and operating conditions. For the fabrication of resonators, suspended transfer of graphene is a common step that might cause damage to the thin suspended membrane. Generally, graphene transfer in micro-mechanical systems is performed with the help of the widely used polymethyl methacrylate (PMMA) [14], Polydimethylsiloxane (PDMS) [15] or other polymers, by spin-coating on the film onto the original substrate surface [16]. Here, we focused on the commonly used transfer method with PMMA substrate. After transferring graphene to a target substrate, PMMA coating could be removed by annealing over 300 °C [17] or washed off in an acetone solution [18]. Considering the PMMA removal method, a previous study by Oshidari et al. [19] has shown that the thermal annealing procedure could be employed to improve the resonator’s resonant frequency and quality factor. By using the Raman spectroscopy imaging technic, it was noticed that there is considerable strain induced in the suspended graphene flakes after annealing, including the furnace annealing [20] or laser annealing [21]. Although this extra inner stress contributes to holding the graphene sheet tightly and causes a flatter membrane surface [22], the extra strain in the annealing process would negatively induce the cracks on the graphene surface. According to Barton et al. [23], the diameter of suspended graphene would affect the performance of the fabricated device. Hence, an appropriate annealing temperature for a graphene membrane with a specific diameter should be evaluated for better performance of the graphene oscillator. Besides the transfer-induced damage in the fabrication process, the photothermal effect would also result in damage to the membrane. Generally, the laser-induced damage can be characterized by the thermal effect and the non-thermal one. For the former, graphene absorbs photons and then releases them under the irradiation of the CW laser [24]. When the applied laser power is strong enough, the energy of phonons can break chemical bonds, thereby resulting in the thermal damage to graphene. The latter could be divided into two aspects. One is due to the original defects, such as the vacancy, which would weaken the fatigue characteristics of the original material, which has been discussed in [7]. The other is due to the ultra-fast energy transfer mechanism unique to solids. When the energy transmission speed of the laser pulse was obviously faster than phonon relaxation time, electrons were excited, and thermions are created. These electrons in semiconductors could absorb energy and then cool down by giving it to other phonons on a shorter time scale than thermal diffusion [25,26]. Melting, vaporization, or sublimation might happen in this stage. Considering our experiment setup, the modulated frequency of the laser was set in the range of 10 kHz to 5 MHz. However, since the phonon relaxation time of graphene is generally in the picosecond order of magnitude [27], which is by far lower than the modulated period of (0.1 μs) the pulsed laser signal in our experiment, this effect mentioned above is not discussed in this paper.

The Raman spectroscopy technique has been used to evaluate the extent of damage by calculating the intensity of the D and G peaks [28]. However, in terms of the actual application, the Raman spectrometer lacks portability. Thus, considering that the surface morphology is suitable for early prediction regarding the resonant state before a resonant test, herein a simple method was developed based on the principle of the Fabry–Pérot (F-P) interference to evaluate the extent of damage through the resonant behaviours of a graphene oscillator. Moreover, it can be seen from the measured resonant response that the thermal time constant acted as a real-time character for monitoring the resonant state of a graphene oscillator.

## 2. Experiment Methods

**Figure 1:**
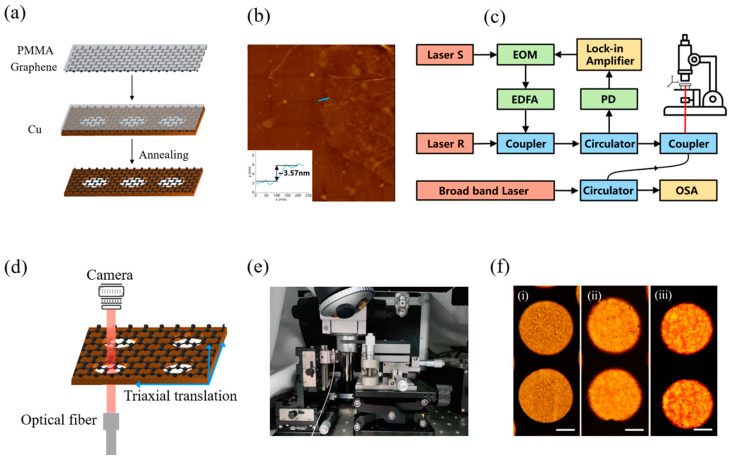
(**a**) The process of making free-standing graphene with Cu substrate, (**b**) the AFM topographic image of a graphene after transfer, (**c**) the experimental setup used to actuate and detect the motion of the resonators, (**d**) the schematic diagram and (**e**) experimental setup of the displacement control, (**f**) the micrographs of 60-μm graphene membranes annealed at (i) 300, (ii) 375 and (iii) 450 °C, scale bar: 20 μm.

Figure 1a shows the process of making free-standing graphene with Cu patterns, wherein a multilayer graphene was grown on the copper (Cu) foil by the chemical vapor deposition. At first, a thin film of PMMA was spin-coated onto the surface of a chemical vapor-deposited multilayer graphene (MLG). The formed MLG/PMMA film was transferred onto the surface of a copper mesh with multiple holes whose diameters were set as 20, 60 and 100 μm, respectively. Figure 1b shows the thickness of the used graphene membrane, which was measured to be 3.57 nm by AFM (FSM Precision, FM-Nanoview 6800, Suzhou, China). The sample with MLG/PMMA film was then placed in a furnace and annealed at a temperature of 300, 375 or 450 °C. Note that the annealing temperature was chosen according to the thermal decomposition characteristic of PMMA [29]. Then, an all-fibre experimental system was established to motivate and interrogate the motion of graphene sheets on the basis of Jin’s work [30], as shown in Figure 1c. In view of a small divergence angle when the laser was irradiated out of the optical fibre into the F-P cavity, the air cavity distance between the fibre and the sample would cause a weak energy loss. In this case, the distance between the membrane and the fibre end-face was controlled to be less than 50 μm via a broad-band laser and an optical spectrum analyser (OSA) on basis of the F-P interference. In order to actuate the graphene membrane, the intensity of laser S was modulated with a rate of 60% and then the membrane was optically heated up and therefore shrank and expanded under the light-induced thermal stress. Then, the opto-mechanics principle in the F-P cavity was employed so as to detect this motion of the graphene, wherein the suspended graphene and the end-face of the optical fibre acted as a moving mirror and a fixed back-mirror, respectively. In this way, the deflection displacement of the suspended graphene membrane could be obtained by a photodetector (PD). For the sake of minimizing the damage caused by excessive laser power, the laser power was set to be as small as possible. To be specific, the light power for laser S and laser R was set to 0.3 mW and 2 mW, respectively. In order to batch test the resonant characteristics of the graphene membrane, the graphene sample without PMMA coating was placed on a precise translation stage with a three-dimension displacement accuracy of 1 μm (Figure 1d,e). In this way, along with the movement observation under a microscope, the light spot of the fibre laser could be adjusted properly in the centre of the membrane. Meanwhile, the photos were read out from computer in real-time, and Figure 1f showed a surface morphology comparison of graphene annealed at different temperatures. The membranes annealed at a higher temperature showed more micro-cracks.

## 3. Results and Discussions

### 3.1. Damage in the Annealing Process

In this section, the damage to the graphene harmonic oscillator after thermal annealing is presented. Graphene membranes annealed at a temperature of 300, 375 or 450 °C were first observed under an optical microscope and scanning electron microscope (SEM) (FEI, Quanta 450 FEG, Reston, VA, USA) to analyse the breakage rate of the membrane, on micro- and nano-scales, respectively. Then, the resonant characteristics, including quality factor and resonant frequency, were investigated by the aforementioned all-fibre experimental system.

**Figure 2:**
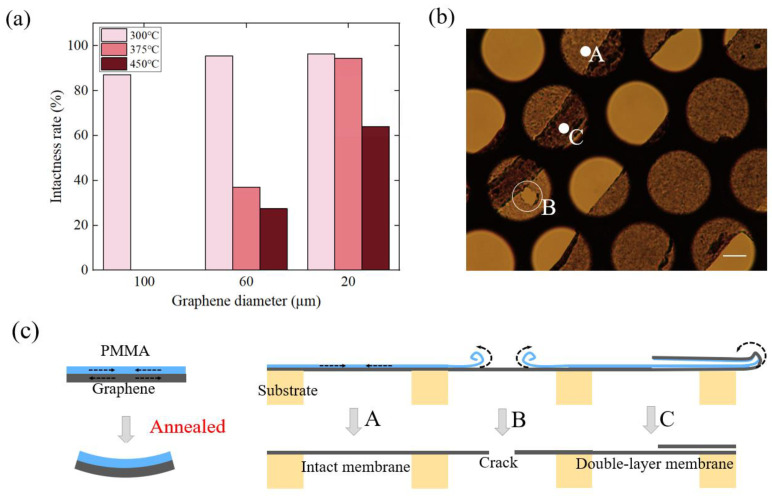
(**a**) The breakage rate of the annealed graphene membrane and (**b**) the microscope photograph of damaged graphene after annealing: points A, B and C represent flat graphene, graphene with a hole and double-layer graphene, respectively. Scale bar: 10 μm, (**c**) the damage mechanism of thermal interfacial interaction, where A, B and C correspond to the regionss A, B and C in Figure 2b, respectively.

After the annealing process at each temperature, the intactness of the graphene was observed by an optical microscope. It has been found that graphene with a more than 95% free-standing area has the potential to be fabricated as a well-behaved harmonic oscillator; thus, this kind of membrane was noted to be intact in this paper. To be specific, 300 graphene membranes after each annealing process were randomly selected, and the number of intact membranes was counted. The statistical results are listed in Figure 2a. Figure 2a shows that the 20 μm-diameter membranes that annealed at 300 °C exhibited the highest rate of intactness at up to 96.4%. Furthermore, graphene membranes were more easily broken with a larger diameter or annealed at a higher temperature, such as 375 °C and 450 °C. This phenomenon could be explained by the stress between PMMA and graphene. In fact, with an increase in the temperature, the decomposition of PMMA coating happened in two stages. In the first stage (at about 220 °C), the C=C bonds of PMMA were broken, while the second stage primarily involved the random scission of C-C bonds at a higher temperature (at about 300 °C) [29]. In view of the mechanical properties of PMMA, the hardness and elastic modulus of PMMA film showed an increasing tendency on account of the reduced chain length of the polymer and cross links of the polymer [31,32]. Therefore, it could be understood that, at a temperature of 300 °C, there was generally a thin film of PMMA left on the graphene surface [33], which kept the membrane rigid and protected the graphene membrane from breaking apart, which is consistent with [23]. However, when the temperature rose above 300 °C, the protection film of PMMA became thinner and left a single membrane of suspended graphene, which was much more easily damaged.

Besides the stress between the graphene and PMMA, there was also a thermal interfacial interaction between the MLG and substrate in the heating process, which would also cause damage to the graphene, especially at the edge of the entire membrane. A photo of a typical damaged graphene caused by thermal interaction between the MLG and substrate is shown in Figure 2b. To be specific, when the resonator was heated, the substrate with a positive thermal expansive efficient would impose tensile stress on the graphene with a negative thermal expansive efficient [34]. Graphene was adhered to the copper surface by the Van der Waals force. So, for some weak points where graphene and the substrate were not fitted closely, the intermolecular force was not strong enough to resist the relative slip between the membrane and the substrate. In this case, the graphene membrane tended to crimp on the metal surface. If the range of the crimp was small, little cracks were found on the graphene membrane, which is marked by point B in Figure 2b. Otherwise, if the crimping range was large, a double-layer membrane was observed, as is shown by point C in Figure 2b. In this case, the thickness of the membrane might double. The corresponding schematic diagram of graphene breakage is shown in Figure 2c. 

**Figure 3:**
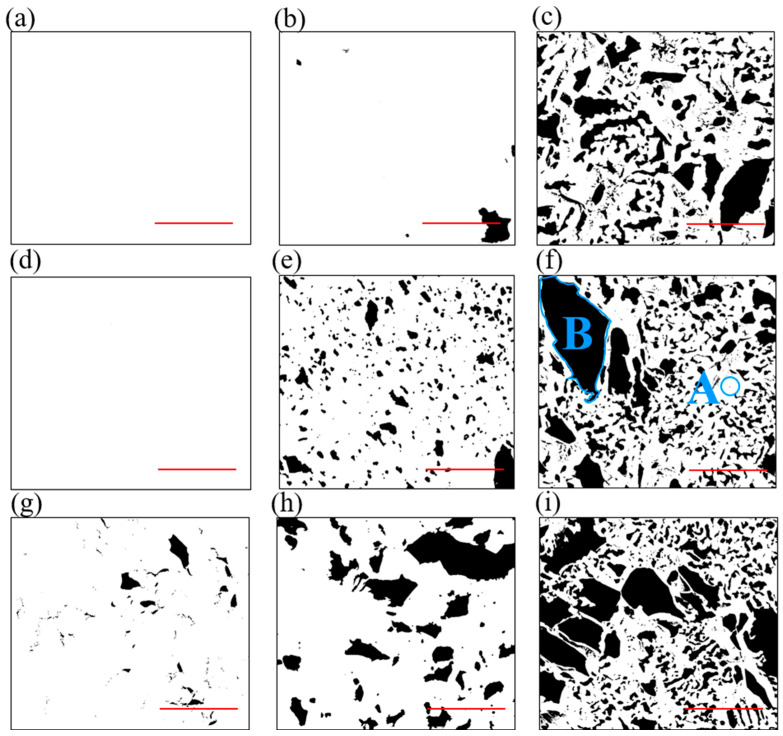
The binarized SEM photograph of graphene of different radius *R* and after annealing at different temperatures *T*, scale bar: 5 μm. The broken rate was noted in the brackets. (**a**) *D* = 20 μm, *T* = 300 °C (0%). (**b**) *D* = 20 μm, *T* = 375 °C (1.6%). (**c**) *D* = 20 μm, *T* = 450 °C (30.9%). (**d**) *D* = 60 μm, *T* = 300 °C (0%). (**e**) *D* = 60 μm, *T* = 375 °C (16.9%). (**f**) *D* = 60 μm, *T* = 450 °C (38.7%). (**g**) *D* = 100 μm, *T* = 300 °C (2.5%). (**h**) *D* = 100 μm, *T* = 375 °C (20.42%). (**i**) *D* = 100 μm, *T* = 450 °C (43.7%).

Furthermore, in order to graphically depict the damage to the graphene membrane on a nano scale, the SEM photographs of the suspended graphene are given in Figure 3. It is worth mentioning that the small amount of PMMA on the graphene surface would lead to a poor image resolution. As a result, gold nanoparticles were sprayed on the PMMA surface to enhance its conductivity. Referring to Figure 3, the dark and bright areas of the image are representative of the broken and the suspended graphene membrane, respectively. For example, in Figure 3f, area A represents the graphene membrane and ‘B’ represents the broken area.

In this way, these SEM images were further binarized to calculate the proportion of the damaged area. Thus, the microscopic broken rate of the membrane could be calculated as num_dark_/num_all,_ wherein num_dark_ and num_all_ represent the number of dark pixels and all pixels, respectively. It could be concluded that a higher annealing temperature *T* or a larger suspended radius *R* would result in a larger broken area. Taking the graphene (*D* = 20 μm, *T* = 300 °C), for example, the graphene exhibited perfect intactness (100%). However, once the suspended diameter was increased to 100 μm, the breakage rate increased to 2.5%. When the annealing temperature was increased to 450 °C, the breakage rate rose rapidly to 30.9%. In the aforementioned experimental set up, the graphene breakage rate showed higher sensitivity to the annealing temperature than the suspended diameter.

**Figure 4:**
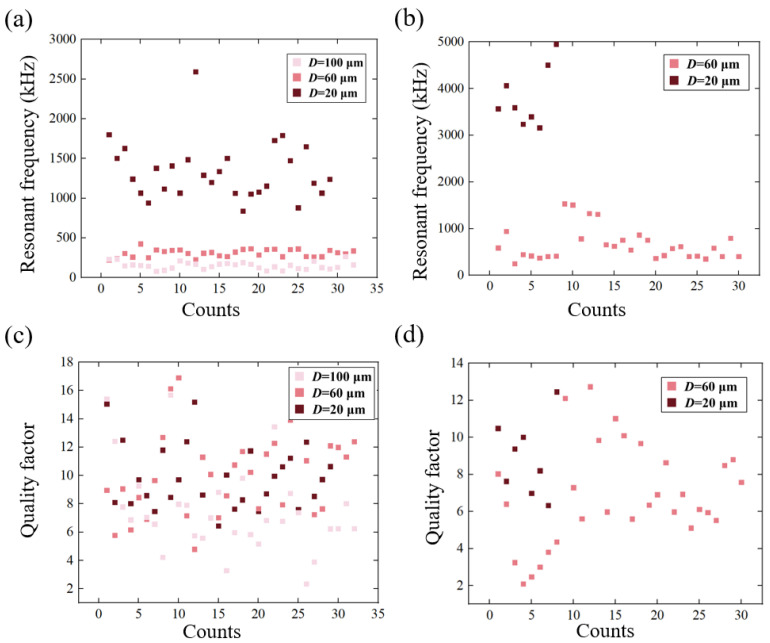
Statistics of resonance frequency and quality factor of graphene at different annealing temperatures and sizes: (**a**) Resonance frequency of graphene annealed at 300 °C; (**b**) the resonant frequency of graphene annealed at 375 °C; (**c**) Quality factor of graphene annealed at 300 °C; (**d**) Quality factor of graphene annealed at 375 °C.

Besides the breakage rate and the surface morphology of the membrane, which has been mentioned above, the resonant characteristics were also investigated, including the resonant frequency and quality factor. Among these, the resonant frequency indicates the fundamental frequency of the oscillator, and the quality factor represents the energy loss per oscillation cycle, which is calculated by *ω*_0_/Δ*ω*, where *ω*_0_ is the natural frequency and Δ*ω* is the 3 dB bandwidth of the amplitude–frequency curve. Thus, the resonant characteristics of the graphene resonators were investigated at the aforementioned three annealing temperatures, as illustrated in Figure 4. 

Figure 4a shows that after annealing at 300 °C, the resonant frequency showed an inverse proportion to the diameter of the graphene membrane. For various graphene films with different diameters, the average frequencies of each 30 samples were respectively calculated as *f*_20_ = 1254.1 kHz (*D* = 20 μm), *f*_60_ = 338.9 kHz (*D* = 60 μm) and *f*_100_ = 153.5 kHz (*D* = 100 μm), and the standard deviation (STD) of frequencies were confirmed as *σ*_20_ = 192.7 kHz (*D* = 20 μm), *σ*_60_ = 59.0 kHz (*D* = 60 μm) and *σ*_100_ = 46.2 kHz (*D* = 100 μm), respectively (Figure 4a). It can be also noticed that the distribution of the resonant frequencies is more concentrated for those resonators with small radii. This is possibly because of the unpredictable breakage of the membrane, which induces more unnecessary vibration modes of the membrane. As for the *Q* factor, the average values were calculated to be *Q*_20_ = 9.8, *Q*_60_ = 9.5 and *Q*_100_ = 7.8, and the STD values were *σ*_20_ = 2.26 (*D* = 20 μm), *σ*_60_ = 2.50 (*D* = 60 μm) and *σ*_100_ = 3.34 (*D* = 100 μm), respectively (Figure 4c). The resonators with smaller diameters had higher *Q* factors and better consistency. Furthermore, these resonators all had a relatively low *Q* factor because the measurement was executed at atmospheric pressure, and the air damping caused high energy loss for the vibrating micro-membrane. In application, the quality factor could be greatly enhanced by sealing the membrane in a vacuum, which could reach an order of thousands.

When the annealing temperature was set as 375 °C, 100 μm-diameter graphene was unable to support itself after annealing. Hence, the resonant data for the 100 μm diameter graphene were not included in Figure 4b. The corresponding average frequencies of the resonators were measured to be *f*_20_ = 3808.5 kHz (*D* = 20 μm) and *f*_60_ = 661.3 kHz (*D* = 60 μm), and the STD values were calculated to be 641.8 kHz and 348.4 kHz, accordingly. The *Q* factors were measured to be 8.93 (*D* = 20 μm) and 6.85 (*D* = 60 μm) with the corresponding STD values of 2.03 and 2.72, respectively (Figure 4d). 

For a circular membrane under tension, the fundamental frequency can be expressed as [23]

(1) $$f=\frac{2.404}{\pi D}\sqrt{\frac{E_{t}S}{\rho \alpha }}$$

where *D*, *E*_t_ and *ρ* are the diameter, the in-plain Young’s modulus and the in-plain density of the graphene, respectively; *S* is the strain in the graphene membrane, and *α* is the density multiplier that describes the contaminating of the device. Moreover, the parameter *ρα* is defined as the in-plain density of the suspended harmonic oscillator including the graphene, PMMA residues and other additional mass. For the resonators at the same annealing temperature, such as 300 °C, graphene membranes were considered to have the same in-plain mass density *ρα*. In terms of Equation (1), the ratio of the inner strain of 20 μm, 60 μm and 100 μm membranes were estimated to be S_1_:S_2_:S_3_ = 2.69:1.78:1. This strain possibly resulted from the Van der Waals force between the graphene and the copper sidewall. At different temperatures, different mechanical energy might be introduced via distortions of the graphene lattice [21]. For the resonators with the same diameter, taking the 20 μm-diameter membrane as an example, it could be inferred that the membrane after 375 °C annealing had three times the resonant frequency compared to the counterparts annealed at 300 °C. Combining the surface appearance depicted in Figure 3, it could be inferred that the inner strain was one of the main factors that caused damage to the membrane.

Note that when the annealing temperature increased to 450 °C, the fabricated resonators of all sizes were damaged with the damaged areas as shown in Figure 2b, and no relative data were recorded in Figure 4. 

### 3.2. Damage in the Laser Irradiation Process

**Figure 5:**
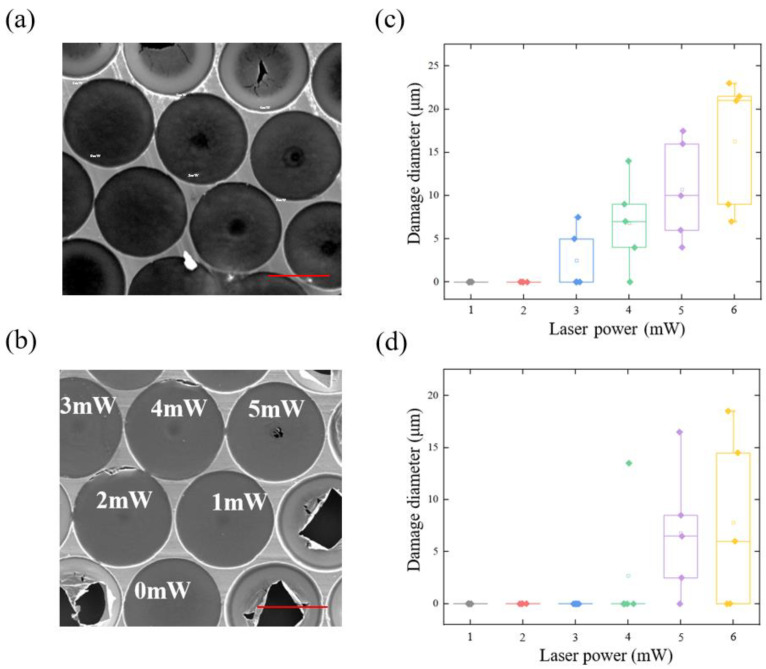
Surface morphology of graphene irradiated by (**a**) modulated laser, scale bar: 40 μm. and (**b**) constant laser (1–5 mW), scale bar: 40 μm. (**c**) damage radius statistics of graphene by modulated pump laser and (**d**) CW laser.

Damage not only occurs in the fabrication process, but also in the working process of the harmonic oscillator. In the previous study on the laser-induced damage, the graphene was often tested by a Raman spectrometer [35] or microscope [36] after laser irradiation, which is ex situ. For a micro-mechanical device, it would be more helpful to perform an in situ detection of the damage situation of the graphene. At this point, the observations in this letter would provide some insight. The 300 °C -annealed 60 μm-diameter graphene membrane was placed under an optical fibre end-face and irradiated by a sinusoidal modulated laser or constant laser. Each membrane was irradiated at a certain laser power for 600 seconds, and the diameter of the damage range was then recorded. Note that the shape of the damage range was sometimes not a strict circle, but an ellipse, in which case the diameter was recorded as half of the sum of the major axis and minor axis of the ellipse. The relationship between the damaged diameter and laser power is shown in Figure 5. Under a modulated laser, cracks were found on the membrane when the power went higher than 2 mW (Figure 5a,c). For the laser with constant power, the membrane centre started to break at a laser power of about 4–5 mW (Figure 5b,d). Note that the fibre optic laser power in our experiment was first measured by a handheld optical power meter (SAMZHE, SZ-GG01, Shenzhen, China) before the sample was irradiated. This could be explained by the fact that a modulated laser would cause not only a heating effect but also alternating photothermal stress within the membrane. The photothermal stress would cause a thermal shock effect on the membrane and accelerate the damage of the membrane [37]. 

**Figure 6:**
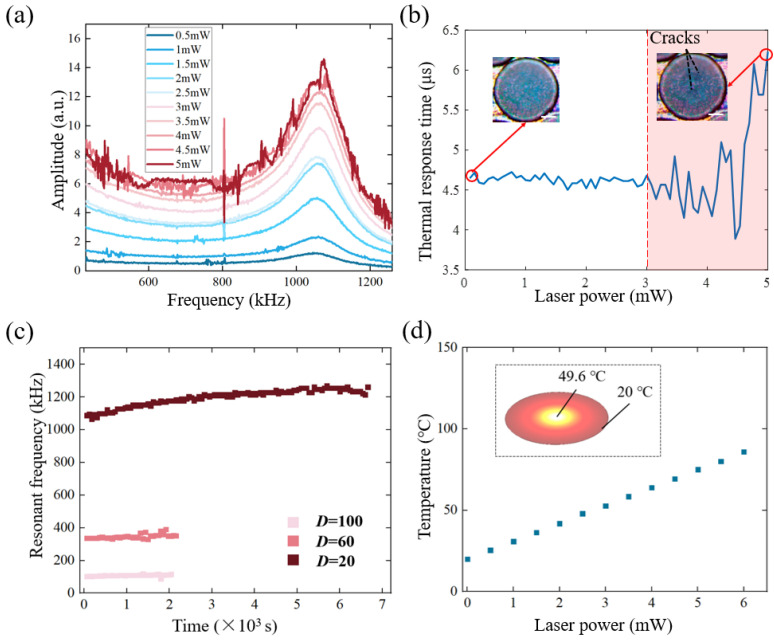
(**a**) The amplitude-frequency response changes when the excitation optical power increases from 0.5 mW to 5 mW. (**b**) The thermal time constant of the resonator changes when the excitation optical power increases from 1 mW to 5 mW; inset: the surface morphology of graphene. (**c**) The long-term static dwelling of graphene with different diameters. (**d**) The calculated temperature under a gaussian laser spot for graphene with three different diameters (thermal conductivity *κ* = 500 W/(mK)); inset: the temperature distribution of the surface.

As the modulated laser was verified with a higher possibility of damaging the membrane, the effect of the modulated laser on the graphene resonant characteristics was further explored. Thus, the graphene membrane was excited with a modulated laser whose power was gradually increased from 1 mW to 5 mW. Meanwhile, the motion was recorded by a CW laser with an extremely small amount of power, so that this laser would barely damage the structure of the graphene. The experiment results were recorded in Figure 6. 

From the frequency domain, Figure 6a shows that the deflection of the oscillator would first increase when the exciting laser power went up. With a further increase of the excitation optical power, the burr of the amplitude–frequency curves increased. This was because the frequency sweep needed to take about tens of seconds, during which the unstable state of the graphene exhibited a fluctuation of the reflected signal. When the laser power finally exceeded 5 mW, a hole was found in the centre of the membrane and no resonance phenomenon could be recorded anymore. 

According to Metzger et al. [38], a thermomechanical response of the suspended graphene could be gained from the frequency domain feature to characterize its thermal properties. One important parameter is the thermal time constant, which describes the response time between the mechanical motion response of suspended graphene and the laser irradiation that opto-thermally actuates the membrane. According to the heat transfer theory, the process of laser irradiation onto the graphene membrane can be considered as the presence of an internal heat source. Combining the optical self-cooling of the deformable Fabry–Perot cavity, the displacement of membrane *z* in the frequency domain could be written as [39]:

(2) $$z(\omega )=\alpha PR\frac{1-i\omega \tau }{1+\omega^{2}\tau^{2}}$$

where *τ* is the thermal time constant, *R* is the thermal resistance, *C* is the thermal capacitance (*RC* = *τ*), *α* is an effective thermal-expansion coefficient and *P* is the heating power. After taking the derivative of Formula (2), the imaginary part of the response function reached the maximum amplitude when *ωτ* = 1. Thus, the thermal time constant *τ* of the graphene membrane was calculated. 

It was found that *τ* started to deviate at about 3 mW (Figure 6b) and then exhibited large fluctuation. The same was the case when the real-time surface morphology started to collapse (Figure 6c), which indicated that the thermal time constant could be a parameter to evaluate the vibration state of the graphene. After that, the thermal time constant went up to about 6 μs from 4.7 μs, which meant a longer time between the actuation and the motion for a broken graphene membrane. 

Then, long-term stability was considered. It was found that the graphene with a smaller diameter tended to have a longer working duration (Figure 6c, blue squares). Combining the surface appearance in Figure 6, there is a considerable possibility that the initial rate of the damage area would have a directly negative effect on the long-term static dwelling of the graphene resonators. Thus, the fabrication method of the lossless graphene membrane is a vital step for applications of graphene resonators and is worthy of further investigation. Moreover, the temperature increase of the graphene sample under a Gaussian beam was simulated with Comsol software with multi-physics fields. The simulation result showed that the thermal effect would not lead to a serious break of the C-C bonds under this laser power [40,41]. That is, it was more likely that the mechanical vibration accelerated this damage. 

## 4. Conclusions

From the perspective of the application of graphene resonant sensors, the effect of the temperature-dependent annealing treatment on the intactness of suspended multilayer graphene was investigated through surface morphology observation to further evaluate the resonant behaviours of graphene resonators after the thermal annealing process. The experimental results showed that an annealing temperature of 300 °C leaves a certain degree of PMMA residue, which can prevent the breakage of graphene while annealing. In contrast, when the annealing temperature rose to 375 °C, more cracks, or even a total collapse, occur in the suspended membrane. In this way, although resonant frequencies that are twice as high could be achieved, the atmosphere pressure quality factor (*Q*_20_ = 8.9) of the resonator showed no synchronous improvement compared to the counterparts annealed at 300 °C (*Q*_20_ = 9.8).

Besides the annealing treatment, the effect of the laser irradiation on the intactness of the suspended multilayer graphene was also investigated. The damage was mainly caused by the modulated laser, which would induce both a thermal effect and mechanical fatigue. The damage threshold power for the modulated laser was found to be about 2–3 mW, which is about half the CW laser. Thus, the modulated laser power should be controlled carefully in application. Moreover, it was found that the fluctuation of the thermal time constant could be applied to evaluate this damage in situ and in real time.

## Data Availability

The data presented in this study are available on request from the corresponding author.
